# Path Planning of Laser Soldering System Based on Intelligent Algorithm

**DOI:** 10.3390/s22218120

**Published:** 2022-10-24

**Authors:** Cong Zhang, Zige Fan, Yaonan Dai, Hang Chen, Sikai Wang, Xubing Chen

**Affiliations:** 1 School of Mechanical and Electrical Engineering, Wuhan Institute of Technology, Wuhan 430205, China; 2 Hubei Provincial Engineering Technology Research Center of Green Chemical Equipment, School of Mechanical and Electrical Engineering, Wuhan Institute of Technology, Wuhan 430205, China

**Keywords:** laser soldering system, improved clustering algorithm, hybrid optimization algorithm, path planning

## Abstract

Laser soldering has been gradually applied to the soldering of electronic components due to the rapid development of microelectronics. However, it is inefficient to use a mechanical shaft to move a laser beam. Here, a laser soldering system is constructed using galvanometer scanning, and an intelligent algorithm is also introduced to optimize the soldering path. Firstly, a laser soldering system for scanning of galvanometers is established, and the functions of visual monitoring, motion planning and parameter integration are presented. Secondly, the position of the laser beam and the corresponding soldering spot are determined, and the coordinate information is provided to plan a route by camera calibration and coordinate system transformation. Finally, the problem of path planning in this system is decomposed into the generation of the soldering point full coverage processing frame, and the route optimization of processing platform and laser beam motion. Furthermore, an improved clustering algorithm, based on the characteristics of system structure, and a hybrid optimization algorithm are designed to deal with the generation of the soldering point full coverage processing frame, the route optimization of processing platform and laser beam motion. In addition, the simulations and experiments are verified by test board. These findings shown that the established system and designed optimization algorithm can promote the efficiency of laser soldering.

## 1. Introduction

With the rapid development of the electronics industry, electronic components are gradually becoming more precise and miniaturized; this development trend is inseparable from the advancement of electronic assembly technology. Electronic assembly technology is based on the design requirements of electronic products through connection technology and auxiliary methods. The soldering process is one of the key links in the electronic assembly process, and the soldering quality will directly affect the quality of electronic products.

Traditional electronic component soldering mainly includes soldering iron, wave soldering, reflow soldering and other methods [1]. However, these electronic assembly methods can interfere with the surrounding components, resulting in a significantly insufficient soldering yield, especially for microelectronic components. Compared with traditional soldering methods, laser soldering has the advantages of non-contact processing, high efficiency, and less interference with surrounding components, so it is widely used in microelectronic components [2]. However, the existing laser soldering system mainly uses mechanical devices to drive the moving position of the laser head, which leads to slow moving speed and low soldering efficiency of the laser head. Compared with traditional soldering equipment, the galvanometer scanning laser soldering system has attracted much attention because of its advantages, such as fast soldering speed, small workpiece stress, small deformation and small heat affected zone [3].

The optical vibrating mirror is a high-speed vector scanner with a single structure tube, small volume, high positioning accuracy and extremely fast scanning speed (MS level). In recent years, optical vibrating mirrors have been widely used in laser soldering. The vibrating mirror is deflected by the motor drive board, and the computer converts the position digital signal into an output analog signal through the D/A card, thereby tracking the propagation of the laser beam.

Currently, the commonly used galvanometer scanning laser soldering system is mainly provided by SCANLAB, GSI, RAYLASE and other manufacturers. Zae et al. [4] proposed a general correction algorithm to solve the distortion problem of the galvanometer scanning pattern and verified that the algorithm has a good correction effect. Chen et al. [5] studied the influence of oscillation frequency and amplitude on the formation of aluminum alloy welds in galvanometer scanning soldering and obtained the optimal weld shape when the frequency was 200 Hz and the amplitude was 0.4–1.2 mm. Li et al. [6] proposed a 3D scanning measurement method to obtain the 3D model information of the workpiece. This method realized the integration of machining and measurement through in situ laser machining and thus reduced the running time and labor cost. Cheng et al. [7] successfully welded aluminum-copper dissimilar metals using nanosecond laser and optical galvanometer scanning. However, the research direction of the existing galvanometer scanning laser soldering system is mainly reflected in improving the soldering accuracy, and the selection of the soldering spot is still carried out by manually selecting the spot or importing the coordinate file of the soldering spot. When the number of solder joints is large, the manual selection method has the problem of heavy workload and low efficiency, and the order of solder joints imported from the coordinate file is not necessarily the optimal soldering path, which also reduces the overall processing efficiency.

Therefore, it is necessary to improve the existing galvanometer scanning laser soldering system to ensure the soldering accuracy and optimize the soldering path. In view of the research on soldering path optimization algorithms, Zhu et al. [8] studied the influence of scanning path on the weld formation of galvanic scanning laser soldering. Tong et al. [9] used the exchange operator and exchange sequence in the particle swarm optimization algorithm to apply the improved algorithm to the path planning of soldering robots. Wang et al. [10] proposed a clustering guided multi-objective particle swarm optimization algorithm and applied it to path planning of spot soldering robots. Based on feature extraction and machine learning algorithms, Chen et al. [11] proposed an online quality monitoring model and defect classification method to realize automatic detection and classification of soldering defects. Based on the data of the laser profiler and the geometric adaptive calculation algorithm, Zaw et al. [12] proposed a positioning method for the soldering torch trajectory. Xu et al. [13] established the 3D point cloud segmentation model of air rudders through a convolutional neural network and obtained the exact soldering path of air rudders. Yang et al. [14] proposed a 3D weld extraction algorithm based on point cloud segmentation to achieve the 3D robust extraction of different welds. Yang et al. [15] proposed a weld bead detection and localization algorithm based on a deep convolutional neural network to achieve accurate detection and localization of weld beads. Hu et al. [16] used the two-level clustering method to ensure that the horizontal weld and the shape appearance were in the same position. Hong et al. [17] used an isometric approximation model to implement path planning of the robot. Zhou et al. [18] proposed an improved discrete MOEA/D (DMOEA/D-HES) parallel scheme based on hybrid environment selection to obtain the optimal order and orientation of searching welds.

The literature [8,9,10,11,12,13,14,15,16,17,18] shows that the length of the machining path in the laser soldering system directly affects the overall machining efficiency, and the overall machining efficiency of the system can be improved by reasonable planning of the machining path. The common laser machining path optimization problem can be regarded as the Traveling Salesman Problem (TSP) [19,20], and the machining efficiency of the system can be improved by solving the shortest machining path. Due to the limited range of laser scanning through the galvanometer, when the processed solder joints of the circuit board exceed the scanning range of the galvanometer, the bottom moving platform needs to be moved to make the solder joints appear in the scanning range of the galvanometer. Therefore, in the whole soldering process, the galvanometer scanning laser soldering system has two moving path problems: a laser is pressure gauge through processing frame private problem of the optimal path of scanning movement, and a laser through movement axis in the frame to move between the machining path optimization problem. These two problems can be regarded as solving the optimal path of the TSP.

In summary, the main contributions of this paper are as follows:

(1) Based on the laser beam deflection and galvanometer intelligent algorithm, the galvanometer scanning laser soldering system is improved, and fast movement of the system is realized by optimizing the soldering path;

(2) According to the calibration position, an improved clustering algorithm is proposed, and the position of the laser beam and the corresponding soldering spot are determined by the active calibration method, which solves the problem of generating the soldering spot full coverage processing frame;

(3) On the basis of the ant colony algorithm and particle swarm optimization algorithm, the hybrid algorithm is used to realize the optimal path selection of the soldering system.

The rest of the paper is organized as follows. In Section 2, the movement path modeling problem of the galvanometer scanning laser soldering system is analyzed. In Section 3, the constructed galvanometer scanning laser soldering system and its calibration method are introduced, and the improved K-means clustering algorithm and hybrid path optimization algorithm are proposed. In Section 4, the improved laser soldering path planning algorithm is simulated and verified by experiments. The conclusion of this paper is in Section 5.

## 2. Path Planning Modeling Principles

The path planned for galvanometer scanning laser soldering should ensure that the laser reaches all solder joints, and at the same time ensure the shortest path of the laser and motion path of the bottom platform.

Aiming at the problems in the soldering process of the traditional tin welder (the iron tip and wire feeding device occupy a large space, the whole plate is heated when heating), the equipment makes use of a laser tin soldering wire feeding device with laser heating characteristics through the temperature control laser tin soldering system, to greatly reduce the occupation of space. At the same time, the controlled-temperature laser soldering system is used for local heating, and non-contact soldering is used to reduce the pressure of soldering parts. Since the scanning speed of the galvanometer is much faster than the moving speed of the bottom processing platform, it is necessary to scan and move the galvanometer as much as possible during the moving process to reduce the number of times the processing platform moves. The bottom machining platform moves on the X and Y axes, and all solder joints are completed by the platform movement.

**Figure 1:**
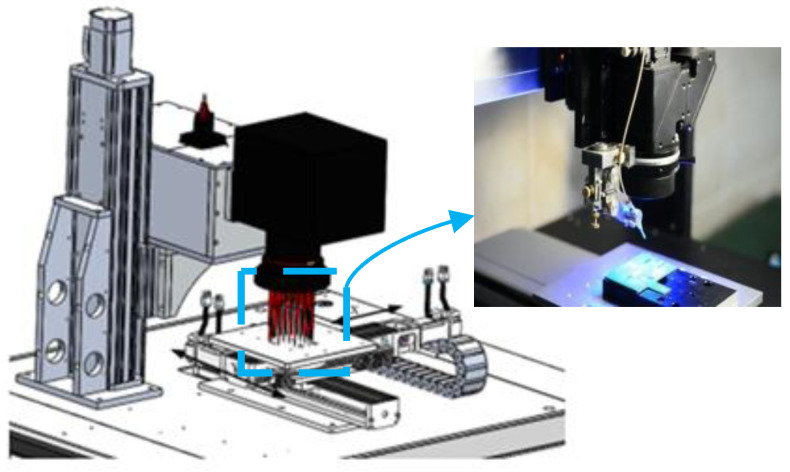
Schematic diagram of system movement.

The schematic diagram of the system movement is shown in Figure 1.

As shown in Figure 1, the workpiece in the galvanometer scanning laser soldering system takes the circuit board as the base plate, and the related electronic components are assembled on the base plate according to the design requirements. In the whole soldering process, the pads irradiated by the laser on PCB are preheated, welded and cooled to realize spot soldering of the equipment. When the system solders all points of the workpiece, it needs to determine the corresponding soldering point position information and then plan the scanning path of the bottom mobile platform and galvanometer to improve the processing efficiency and soldering effect.

### 2.1. Processing Frame Generation Problem Description

**Figure 2:**
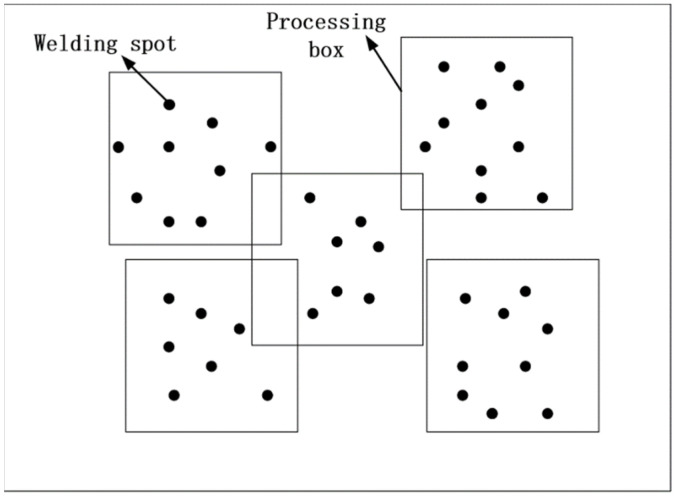
Schematic diagram of full coverage of solder joints.

In the system constructed in this paper, the theoretical processing range of the laser passing through the galvanometer is 50 × 50 mm, and the processing frame represents the frame selection area of the processing range. When the position of the solder joints in the circuit board exceeds the processing range of the galvanometer, it is necessary to plan the corresponding movement axis to move the points to complete the processing coverage of all solder joints. The full coverage of the solder joints of the processing frame is shown in Figure 2.

In the system constructed in this paper, the theoretical processing range of the laser through the galvanometer is 50 × 50 mm, and the processing frame represents the frame selection area of the processing range. When the solder joint position on the circuit board exceeds the scope of the galvanometer, the corresponding motion axis needs to be planned to move the position of the point until all solder joints are covered. The full coverage of the solder joints of the machining frame is shown in Figure 2.

Due to the limited range of laser scanning, when the processing solder joints on the circuit board exceed the scanning range of the galvanometer, the bottom mobile platform needs to be moved to make the solder joints appear within the scanning range of the galvanometer. In order to improve the soldering efficiency, it is necessary to determine the minimum number of processing frames. The whole process is described as follows.

(1) There are *n* solder joints on the circuit board, and the set of points is $C=\{c_{1},c_{2},\cdots c_{n}\}$.

(2) The set of processing clusters is Q={1,⋯,m}, *m* is the number of processing clusters, the number of processing frames is consistent with the number of processing clusters, and the center of processing clusters is the center of the processing frame.

(3) The range of the processing frame should be consistent with the processing range scanned by the laser through the galvanometer. Any solder joint *C_i_* (*x_i_*, *y_i_*) must belong to the processing cluster. At the same time, the processing cluster needs to be smaller than the processing frame size, and the constraints are as follows:

(1) $$\{\begin{array}{c} X_{\max}^{k}-X_{\min}^{k}\leq L_{x}\\ Y_{\max}^{k}-Y_{\min}^{k}\leq L_{y} \end{array}$$

where, $X_{\max}^{k}$ and $X_{\min}^{k}$ represent the maximum and minimum values of the processing cluster *k* on the *X* axis, respectively. $Y_{\max}^{k}$ and $Y_{\min}^{k}$ represent the maximum and minimum values of the processing cluster *k* on the *Y* axis, respectively. *L_x_* and *L_y_* represent the lengths of the machining frame in the *X* axis and *Y* axis directions, respectively.

### 2.2. Moving Path Problem Modeling

When soldering components on a circuit board, the laser beam needs to reach all solder joints. During the whole soldering process, there are two moving paths between the galvanometer scanning laser soldering system and soldering point. In order to make the total distance from the laser beam to the soldering point as short as possible, for different types of circuit board galvanometer scanning systems, path planning needs to be carried out according to the location of different circuit board soldering points to achieve the best path. The process of model construction is described as follows:

(1) There are *N* points in the problem, the walking points are represented as *C_i_* (*x_i_*, *y_i_*), and the set of points is $C=\{c_{1},c_{2},\cdots c_{n}\}$.

(2) A non-repeating permutation order $S=\{s_{1},s_{2},s_{3},\cdots ,s_{N-1}\}$ is found in the set *C*. *S* needs to traverse all points in the set *C* without passing through the repeated points.

(3) The problem can be described as finding a traversal path *S* of the soldering point that satisfies the objective function. The objective function is as follows:

(2) $$f(C)=\min\sum_{i=1}^{N-1}d(c_{i},c_{i+1})$$

## 3. Galvo Scanning Laser Soldering System and Its Path Planning

### 3.1. Galvo Scanning Laser Soldering System

**Figure 3:**
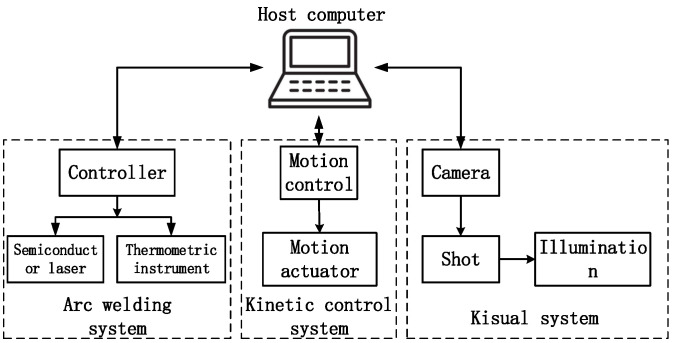
Overall frame diagram of the system.

The galvanometer scanning laser soldering system includes a motion control system, soldering system, vision system and industrial control host computer. The overall frame of the system is shown in Figure 3.

The motion control system mainly controls the movement of the galvanometer lens and the three mechanical axes, controls the movement of the laser and camera through galvanometer scanning, and monitors the field of vision. At the same time, the mechanical axis is used to control the movement of the workpiece and galvanometer lens. The soldering system includes lasers, temperature measurement modules, etc., and completes the soldering tasks according to the instructions issued by the industrial computer.

The vision system is used to obtain the current position information. Through optical design, the vision, laser and infrared are coaxial, so that the soldering position is consistent with the position of the visual monitoring area. The industrial control host is the information processing center of the system, which is mainly responsible for the control of the whole system.

Among them, the camera in the vision system adopts an area scan camera that can obtain images more intuitively. The optical system uses a coaxial lens to make the laser beam, infrared and visual light on the same optical axis. The lens at the optical outlet uses a telecentric lens to correct the parallax caused by the industrial lens. Since the light source has an important impact on the quality of the final captured image, the annular light source with the specification of SN-90(188) is selected in this paper.

**Figure 4:**
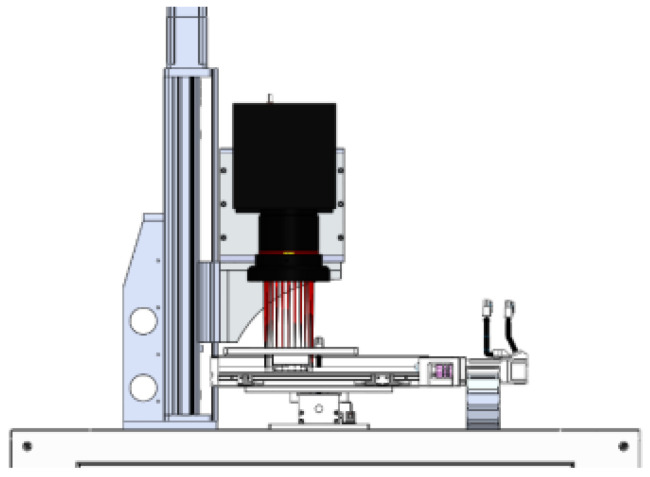
Schematic diagram of the system motion axis structure.

The motion control system includes galvanometer scanning motion control and three-axis motion. Among them, the galvanometer lens in the system adopts a two-dimensional galvanometer, and there are scanning lens X and scanning lens Y inside. The relevant parameters of the galvanometer lens are shown in Table 1. The three-axis motion includes three motion axes, X, Y and Z, among which the X and Y axes are responsible for the movement of the processing platform. The Z axis moves the galvanometer up and down so that it can focus and defocus the laser. The system motion axis structure is shown in Figure 4.

The soldering system includes a semiconductor laser, STM32 microcontroller and a thermometer with a range of 100 to 600 °C. Compared with other types of lasers, semiconductor lasers interact better with metals and are more easily absorbed by them, thus facilitating soldering. At the same time, the semiconductor laser can be coupled with the fiber to facilitate the transmission of the laser beam. Related parameters of semiconductor laser are shown in Table 2.

The controller uses a STM32 microcontroller, which can meet the demand of the thermometer acquisition rate. Through feedback adjustment, the controller can effectively ensure that the temperature on the solder joint is basically constant. The related technical parameters of the thermometer are shown in Table 3.

### 3.2. Camera and Workpiece Calibration Research

(1) Calibration of the processing position of the galvanometer

In the galvanometer laser scanning system, the laser beam is shifted by rotating X, Y and two galvanometers. In the actual machining process, it is necessary to determine the distance conversion relationship between the input position value of the vibrator scan and the actual optical output position. The distance between the solder joints of the marking plate was measured by electron microscopy. During the marking process, the system gave an interval coordinate value of 5000. In order to reduce the error caused by measurement, the distance between points was measured 20 times in the actual process, and the average distance was calculated. The conversion equation between the calculated coordinate value and the actual distance is

*L*_m_ = 0.001534 × *P_s_* (3)

where *L*_m_ is the actual corresponding distance, and *P_s_* is the input coordinate value.

(2) System camera calibration method

The transformation relationship between the pixel coordinate system and galvanometer processing coordinate system is obtained by calibration in the laser galvanometer scanning system. Since the shooting surface of the camera is not in the same direction as the actual shooting surface, the reflected light path of the telecentric lens and galvanometer is regarded as the processing surface into which the camera shoots vertically.

**Figure 5:**
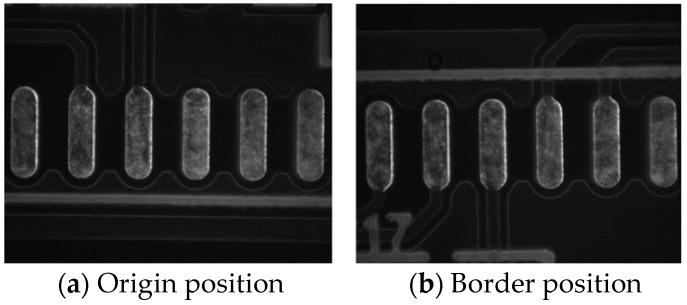
Images of the actual situation.

In the galvanometer laser scanning system, the design of the telecentric lens makes the distortion very small. When the galvanometer deflects to the critical point, the maximum distortion is 0.5283%, and the scanning range of the galvanometer can meet the soldering use of the system. Images taken at different positions are shown in Figure 5.

Comparing the boundary position image captured by the camera with the origin image, it can be found that the distortion produced by the camera is very small.

(3) System camera calibration method

**Figure 6:**
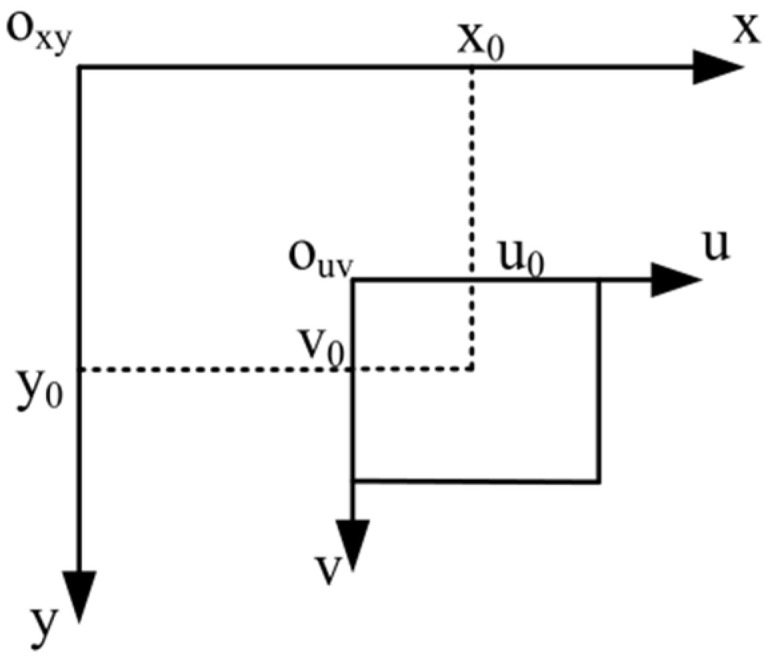
Relationship between the pixel and galvanometer coordinate systems.

Because the laser optical path in the actual system is coaxial with the visual optical path, it can be equivalent to the pixel coordinate system. At the same time, the height of the machining surface and the luminescence point remain unchanged during the machining process, so the machining coordinates of the galvanometer can be converted into two-dimensional coordinates. The camera needs to change its position with the movement of the galvanometer, so the deflection of the galvanometer can be used to carry out the calibration process. The relationship between the pixel coordinate system and galvanometer processing coordinate system is shown in Figure 6.

In the figure, ($x_{0},y_{0}$) is the deflection position of the galvanometer, and $\left(u_{0},v_{0}\right)$ is the position of the center point of the laser spot in the image when the galvanometer is turned to the position of ($x_{0},y_{0}$). At the same time, when the two coordinate systems are converted, the unit needs to be unified. The transformation relationship between the two coordinate systems is as follows:

(4) $$\left\{ \begin{array}{l} x=x_{0}+w_{x}(u-u_{0})\\ y=y_{0}+w_{y}(v-v_{0}) \end{array}\right.$$

**Figure 7:**
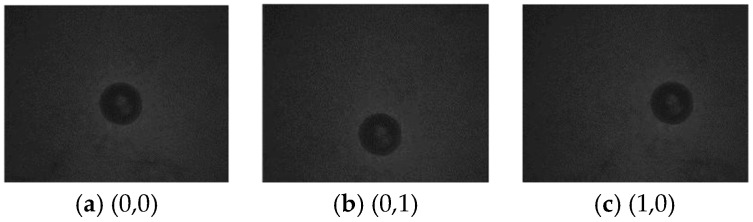
Pictures of marking points taken at different positions.

The system is used to mark a point on the marker plate when the galvanometer is deflected towards the origin. The pixel position of the center of the circle in the image is identified by moving the galvanometer, and the corresponding galvanometer motion position is recorded. The motion of galvanometer makes the camera shoot at (0,0), (1,0) and (0,1), as shown in Figure 7. The conversion relationship between the galvanometer coordinate system and the pixel coordinate system is calculated through three points, and the calculation results are as follows:

(5) $$\left\{ \begin{array}{l} x=x_{0}+0.0056(u-668.5)\\ y=y_{0}+0.0056(v-568.5) \end{array}\right.$$

**Figure 8:**
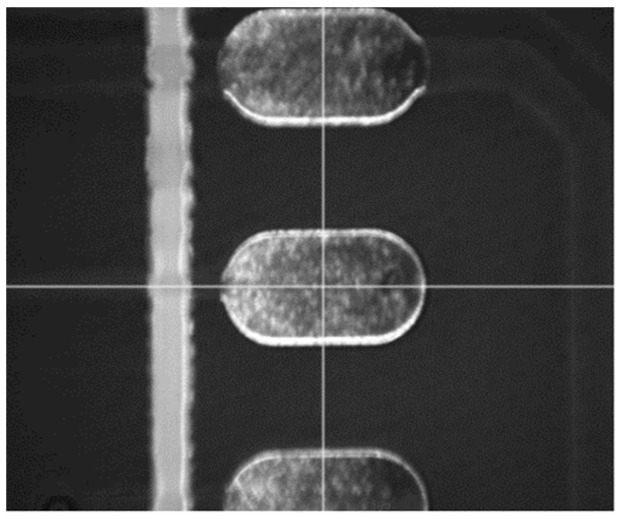
Display of the light output position.

After the actual measurement of the conversion relationship between the laser luminescence position and the image coordinate system, a cross is drawn to mark the luminescence position in the image display area, to facilitate subsequent operations. The light output position is shown in Figure 8.

### 3.3. Workpiece Position Calibration

Before machining the system, the solder joint position information of the workpiece needs to be imported into the system. After setting the origin coordinates in the PCB source file, location information such as marker points and pad positions can be exported in Altium Designer software.

In the actual machining process, the placement position of the workpiece is not completely parallel to the machining plane, so it is necessary to calculate the mark points on the circuit board in the system and calculate the transformation relationship between the workpiece coordinate system and the machining coordinate system of the oscillator meter [21]. At the same time, according to the position of the marker in the pixel coordinate system, and the transformation relationship between the pixel coordinate system and the galvanometer processing coordinate system, the relationship between the solder joint position and the galvanometer processing coordinate system can be obtained.

(1) Marking point recognition

**Figure 9:**
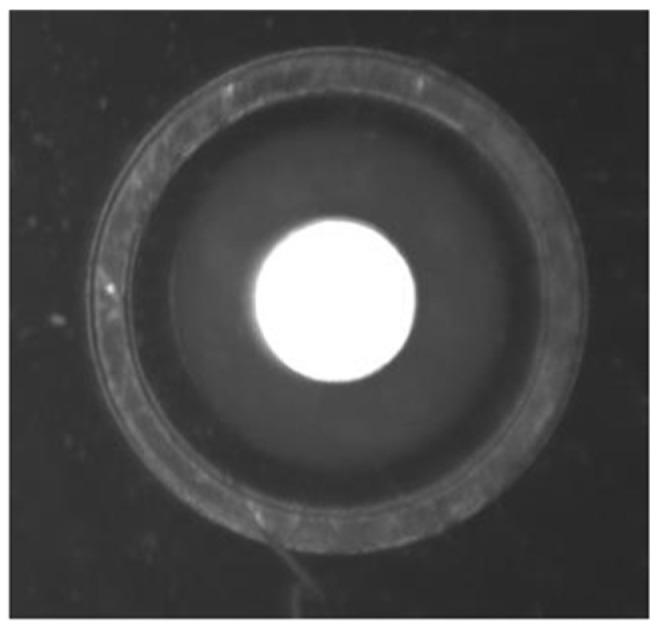
Marking point setting style of circuit board.

**Figure 10:**
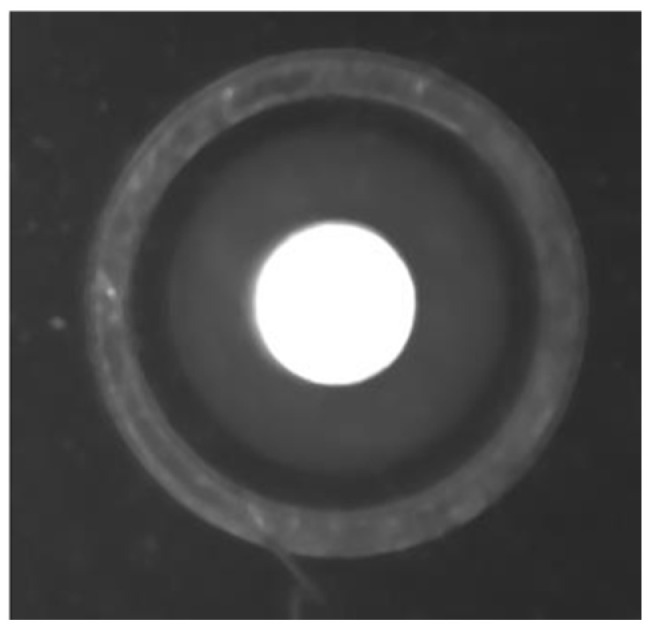
Median Filtering.

**Figure 11:**
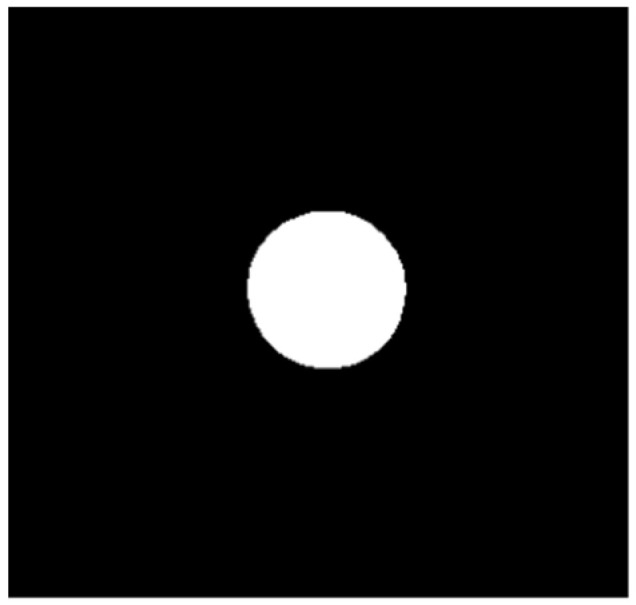
Image after binarization.

**Figure 12:**
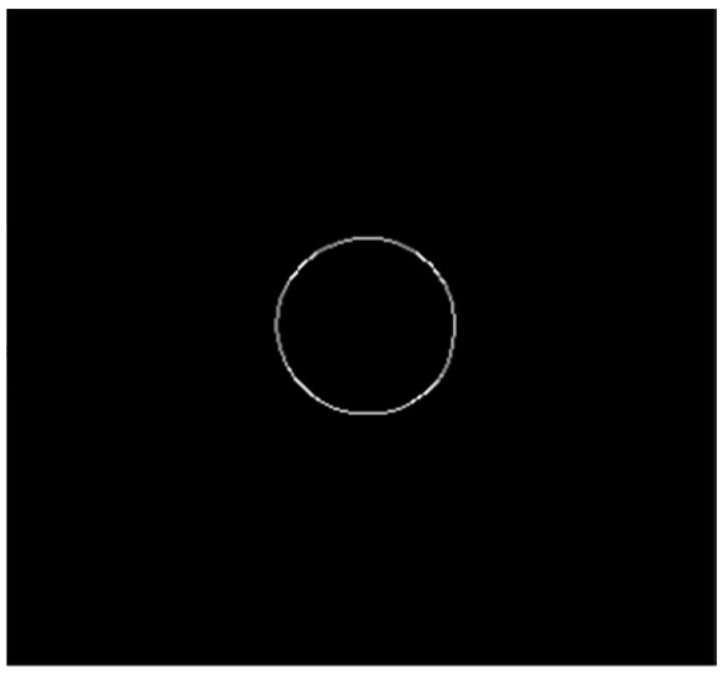
Pixel edge extraction.

Common marking points on the circuit board include circles, crosshairs and other figures. The setting diagram of marking points on the circuit board in this paper is shown in Figure 9. In the actual detection process, the marker is usually set far away, and the center of the marker is asymmetric. Therefore, the image acquired by the camera should be preprocessed, and the noise on the image should be eliminated by median filtering. The median filtered image is shown in Figure 10. After removing the noise, the image is binarized and segmented. The image after binarization is shown in Figure 11. The pixel edge points in the binarized image are extracted, and the extracted image is shown in Figure 12. Since the image after point imaging will be an ellipse rather than an ideal circle, circle fitting is required.

After the pixel edges are obtained, the least squares fitting is performed on the obtained pixel edge points. The least squares circle fitting equation is

(6) $$r^{2}={\left(x-A\right)}^{2}+{\left(y-B\right)}^{2}$$

where *r* is the radius of the circle, and the point (*A*, *B*) is the center of the circle. Another form of the fitted circle equation can be obtained by making $a=-2A,\;b=-2B,\;c=A^{2}+B^{2}-r^{2}$:

(7) $$x^{2}+y^{2}+ax+by+c=0$$

According to the principle of least squares, the objective function can be obtained as

(8) $$Q(a,b,c)=\sum_{i=1}^{N}{\left(x_{i}{}^{2}+y_{i}^{2}+ax_{i}+by_{i}+c\right)}^{2}$$

When the objective function takes the minimum value, the fitting accuracy is the best. To make the objective function reach the minimum value, it can be obtained according to the extreme value principle:

(9) $$\frac{\delta Q}{\delta a}=\frac{\delta Q}{\delta b}=\frac{\delta Q}{\delta c}=0$$

where the values of *a*, *b*, and *c* can be solved. At this time, the radius of the circle and the position of the center of the circle are

(10) $$\left\{ \begin{array}{l} r=\frac{\sqrt{\left(a^{2}+b^{2}-4c\right)}}{2}\\ \left(-\frac{a}{2},-\frac{b}{2}\right) \end{array}\right.$$

**Figure 13:**
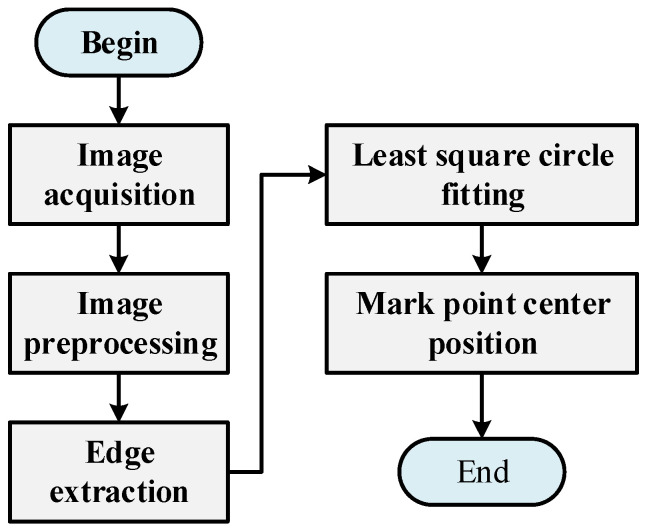
Visual processing flow.

The visual processing flow is shown in Figure 13.

(2) Processing coordinate conversion

**Figure 14:**
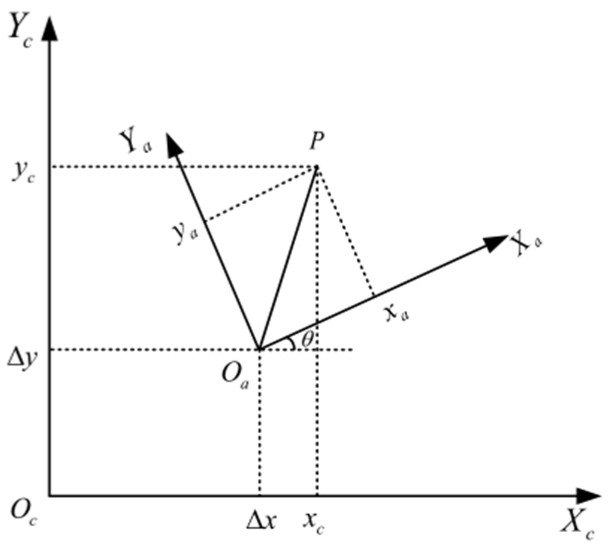
Coordinate system conversion relationship.

During the processing of the system, the position of the solder joints directly imported through the Altium Designer software needs to be converted into the galvanometer processing coordinate system, so it is necessary to use the imaging point for calibration process to complete the transformation of the position relationship. The coordinate system conversion relationship between the galvanometer processing coordinate system and the actual workpiece is shown in Figure 14.

$X_{c}O_{c}Y_{c}$ is the coordinate system for galvanometer processing, and $X_{a}O_{a}Y_{a}$ is the coordinate system when the actual workpiece is designed. The coordinate of point P in the workpiece coordinate system is set as $\left(x_{a},y_{a}\right)$, and the coordinate in the galvanometer processing coordinate system is $\left(x_{c},y_{c}\right)$; there are rotation and translation between the two coordinate systems, and the transformation relationship is as follows:

(11) $$\left[\begin{array}{l} x_{c}\\ y_{c} \end{array}\right]=[\begin{array}{l} \cos\theta \\ \sin\theta \end{array}\begin{array}{l} -\sin\theta \\ \cos\theta \end{array}]\left[\begin{array}{l} x_{a}\\ y_{a} \end{array}\right]+\left[\begin{array}{l} \Delta x\\ \Delta y \end{array}\right]$$

In the process of position transformation, the actual position corresponding to the center of the circle is obtained by identifying four marking points on the workpiece. At the same time, the coordinate positions of the four marking points on the workpiece are derived by software in the workpiece coordinate system, and the actual positions of the corresponding solder points in the workpiece can be obtained by Equation (10). After the actual position of the circuit board is obtained, the pixel position of the marking point in the image is used to convert the actual solder position into the galvanometer table processing coordinate system according to the conversion relationship between the pixel coordinate system and the galvanometer processing coordinate system.

### 3.4. Systematic Path Planning Improvement Algorithm

(1) Full coverage soldering point processing frame generation

The size of clusters in solving traditional clustering problems is not limited. The number of clusters in the K-means clustering algorithm needs to be determined in advance as the initial value input into the algorithm, and the number of clusters is often obtained through experience. The size of the clusters is limited by the processing range, and there is no limit to the number of clusters; the number of clusters needs to be as small as possible. At this time, the traditional K-means algorithm cannot be directly applied to this problem.

In order to solve the problem of processing frame generation with full coverage of solder joints, the K-means clustering algorithm is improved as follows:

Step 1: Divide the workpiece into meshes; the mesh size is consistent with the size of the processing frame, and each mesh area is used as an initial cluster.

Step 2: Delete the grid that does not contain the position of the solder joint. The position of the solder joint appears on the corresponding position of the circuit board according to the design requirements. If there is no solder joint position in the area, it is an invalid grid.

Step 3: Perform sample allocation for each cluster. During the sample allocation process, the size of the cluster range after adding the sample should not exceed the size of the processing frame.

Step 4: Recalculate the cluster center and adjust the division of the sample data.

Step 5: For each cluster, try to merge the nearby clusters. If the range of the merged clusters does not exceed the size of the processing frame, the two clusters are merged. The generated cluster collection, n, is the number of clusters. Each cluster needs to meet the following requirements:

(12) $$\left\{ \begin{array}{l} R_{x\cdot \max}^{k}-R_{x\cdot \min}^{k}\leq L_{x}\\ R_{y\cdot \max}^{k}-R_{y\cdot \min}^{k}\leq L_{y} \end{array}\right.$$

where $R_{x\cdot \max}^{k}$ and $R_{x\cdot \min}^{k}$ are the maximum and minimum values of cluster *k* in the *X* direction, respectively.$R_{y\cdot \max}^{k}$ and $R_{y\cdot \min}^{k}$ are the maximum and minimum values of cluster *k* in the *Y* direction, respectively. $L_{x}$ and $L_{y}$ represent the lengths of the machining frame in the *X*-axis and *Y*-axis directions, respectively; any of the final generated clusters are within the scope of the processing frame.

Step 6: Repeat steps 3 to 5 until there are no merged clusters.

**Figure 15:**
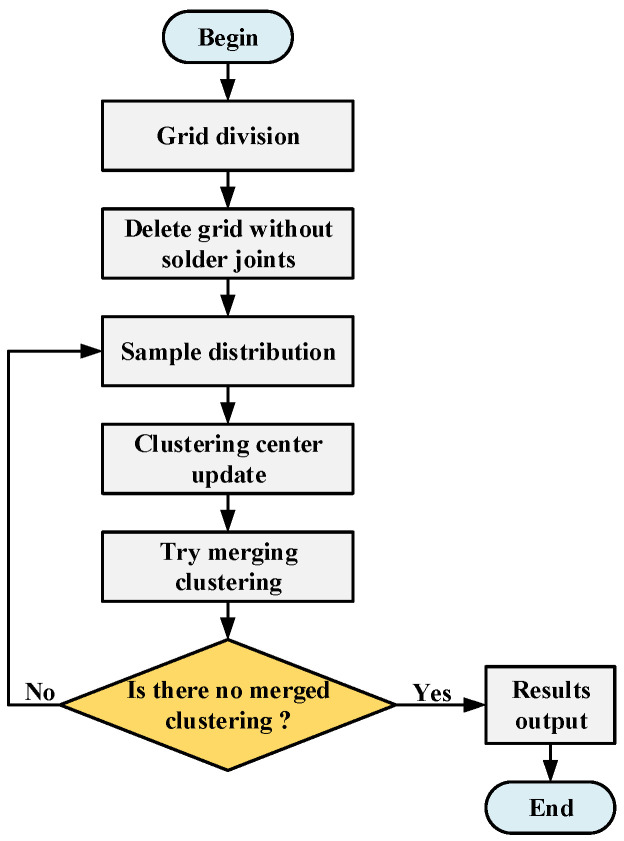
Algorithm full coverage processing frame generation algorithm flow.

The overall algorithm flow is shown in Figure 15.

**Figure 16:**
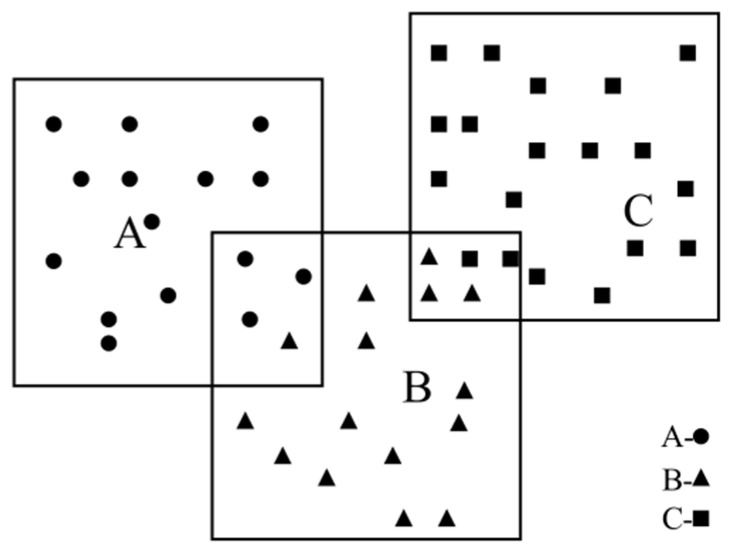
Attribution of solder joints in the intersection part.

There will be intersections between the processing frames obtained by the full coverage solder joint processing frame generation algorithm. For the problem of attribution of solder joints in the intersection part, the position of the processing frame of the solder joints belonging to the cluster has the solder joints in the intersection part. The schematic diagram of the attribution of cross solder joints is shown in Figure 16.

(2) Principle of Ant Colony Algorithm

The ant colony algorithm searches the optimal path by simulating the behavior of ants searching for food. When an ant searches for food, pheromones stay in the path it takes, and the best path is determined based on the concentration of pheromones the ant leaves behind. In the process of finding food, the ant colony determines the next destination by the concentration of pheromones, and its transition probability is as follows:

(13) $$\left\{ \begin{array}{ll} \frac{{\left[\tau_{ij}(t)\right]}^{\alpha }{\left[\eta_{ij}(t)\right]}^{\beta }}{\sum {\left[\tau_{is}(t)\right]}^{\alpha }{\left[\eta_{is}(t)\right]}^{\beta }} & S\in allow_{k}\\ 0 & S\notin allow_{k} \end{array}\right.$$

where *k* is the number of ants in the ant colony, and $\tau_{ij}(t)$ is the pheromone concentration between the two cities at t time. $\eta_{ij}(t)=1/d_{ij}$ is used to represent the heuristic function, and $d_{ij}$ is the distance between two cities. α is the pheromone importance factor, *β* is the heuristic function importance factor; the larger the value, the greater the probability of moving to this point. $allow_{k}$ is the set of cities that ant *k* needs to visit.

When all ants complete a cycle, the pheromone concentration needs to be updated, and the pheromone update is calculated as follows:

(14) $$\left\{ \begin{array}{l} \tau_{ij}(t+1)=(1-\rho )\tau_{ij}(t)+\Delta \tau_{ij}\\ \Delta \tau_{ij}=\sum_{k=1}^{n}\Delta \tau_{ij}{}^{k} \end{array}\right.\;0<\rho <1$$

Among them, ρ is the pheromone volatilization concentration, $\Delta \tau_{ij}$ is the sum of the pheromone concentrations released by all ants between the two cities, and $\Delta \tau_{ij}{}^{k}$ is the information concentration left by the ant k in the two cities.

In the classic ant colony algorithm, $\Delta \tau_{ij}{}^{k}$ is usually calculated as

(15) $$\Delta \tau_{ij}{}^{k}=\left\{ \begin{array}{l} \frac{Q}{L_{K}},\;\mathrm{The}\;k\text{-}\mathrm{th}\;\mathrm{ant}\;\mathrm{traverses}\;\mathrm{the}\;\mathrm{path}\;\mathrm{in}\;\mathrm{this}\;\mathrm{loop}\;(i,j)\\ 0,\;\mathrm{otherwise} \end{array}\right.$$

where Q is a constant, and *L_k_* is the total length of the path passed by the *k*-th ant.

The ant colony algorithm needs to set the parameters in advance, and the parameter setting has a great influence on the final effect of the algorithm.

Pheromone heuristic factor α: The value of the pheromone heuristic factor is too small. The blindness of the ant’s path search is relatively large, and the convergence speed of the algorithm will be slowed down. When the value of the pheromone heuristic factor is too large, the algorithm will easily converge prematurely and fall into the local optimal solution.

Expectation heuristic factor *β*: When the value of *β* is too large, the ants are more likely to choose the local optimal path. When the value of *β* is too small, the randomness of ants searching for the optimal path is weakened, and it is easy to fall into a local optimum.

(3) Path optimization based on hybrid algorithm

The ant colony algorithm can find feasible solutions in a short time and can also combine the advantages of other algorithms to improve the overall performance of the algorithm. However, when the scale of the problem becomes larger, the ant colony algorithm tends to fall into the local optimal solution, and the convergence speed slows down [22]. Particle swarm optimization (PSO) is a random search algorithm proposed by simulating bird foraging. The particle swarm algorithm has strong local search ability in the early stage. The initial distribution of pheromones in ant colony algorithm is set by the particle swarm optimization algorithm, which can effectively solve the problem of low search efficiency in the initial case of the ant colony algorithm. According to this characteristic, the combination of particle swarm optimization and the ant colony algorithm can improve the effect of path optimization.

In the galvanometer scanning laser soldering system, the processing platform and the moving path of the laser beam do not need to return to the starting point, and the algorithm needs to be modified accordingly. The working steps of the overall hybrid algorithm are as follows:

Step 1: Initialize the parameters related to the particle swarm, including the number of particles, learning factors, inertia factors and other parameters.

Step 2: Randomly generate the initial position and velocity of particles, calculate the fitness value of each particle, and calculate the initial individual extremum and group extremum. Add virtual points. The virtual points are used as the start and end points of the path. At the same time, the distance from the virtual point to any point is set to 0.

Step 3: Update the inertia factor; the update equation is shown as follows:

(16) $$w=w_{\max}-(w_{\max}-w_{\min})\frac{k}{M}$$

where w is the inertia factor, $w_{\max}$ is the maximum inertia factor, $w_{\min}$ is the minimum inertia factor, k is the current iteration number, and M is the overall iteration number of the particle swarm algorithm.

Step 4: Update the individual extremum and the global extremum of each particle. If the fitness value is greater than the individual extremum, replace the fitness value with the individual extremum. If the fitness value is greater than the global extreme value, the fitness value is replaced by the global extreme value.

Step 5: Each particle updates its velocity and position according to its own individual optimal value and the global optimal value of the particle swarm. Update the particle’s velocity and position according to the update equation. The update equation is as follows:

(17) $$\left\{ \begin{array}{l} v_{id}(t+1)=wv_{id}(t)+c_{1}r_{1}(p_{id}-x_{id}(t))+c_{2}r_{2}(p_{gd}-x_{id}(t))\\ x_{id}(t+1)=x_{id}(t)+v_{id}(t+1) \end{array}\right.$$

where $v_{id}$ is the velocity of the particle i in the dth dimension, and $x_{id}(t)$ is the position of the particle i in the dth dimension. $c_{1}$ and $c_{2}$ are learning factors, which reflect the particle’s self-learning ability and the learning ability of the group optimal particle, respectively. $r_{1}$ and $r_{2}$ are uniform random numbers. $p_{id}$ is the individual extreme value, and $p_{gd}$ is the global extreme value.

Step 6: Repeat steps 3–5 and stop when the set maximum number of iterations is reached.

Step 7: Carry out the initial distribution of the ant colony pheromone according to the results of the preliminary planning. Let the initial pheromone value of the ant colony algorithm be $\tau_{ij}^{s}$, and the pheromone converted by the particle swarm algorithm after path planning be $\Delta \tau_{ij}^{pso}$; at this time, the initial pheromone of the ant colony algorithm becomes

(18) $$\tau_{ij}=\tau_{ij}^{s}+\Delta \tau_{ij}^{pso}$$

Step 8: Add a virtual point to the ant colony algorithm; the position of the virtual point to any point is 0, and the start and end points of the path are virtual points.

Step 9: When the ant colony algorithm reaches the maximum number of iterations, it stops and outputs the optimized path result. At this time, the starting point of the path is the virtual point as the starting point and the next point, and the end point is the virtual point as the end point and the previous point.

**Figure 17:**
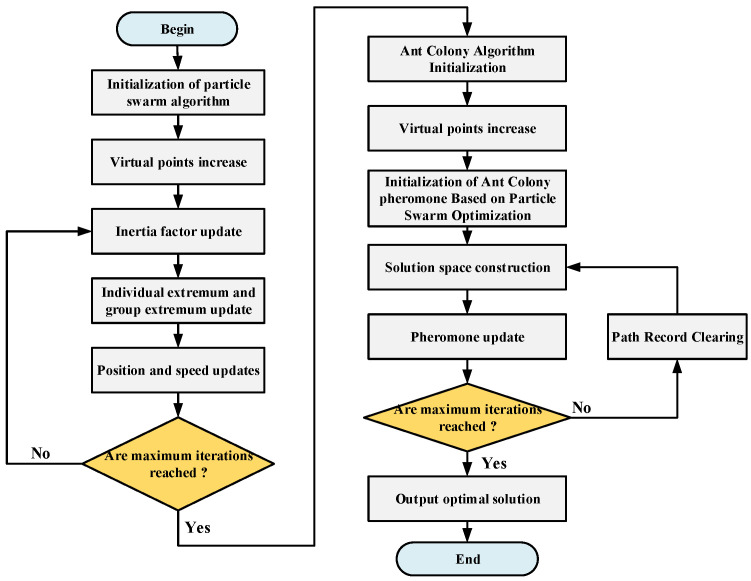
Flowchart of the hybrid algorithm.

The workflow of the hybrid algorithm is shown in Figure 17.

## 4. Path Planning Analysis

### 4.1. Generation of Machining Frameworks

**Figure 18:**
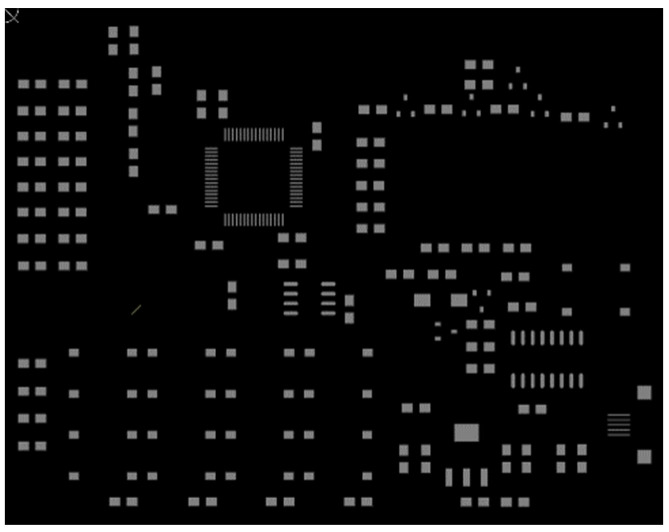
Distribution of solder joints on the circuit board.

The improved K-means clustering algorithm is used to generate a processing frame for solder joints in circuit boards, which requires that the smallest processing frame can completely cover the solder joints in circuit boards. A sample with a circuit board size of 87 mm × 68 mm is selected for testing. The distribution of solder joints on the circuit board is shown in Figure 18.

**Figure 19:**
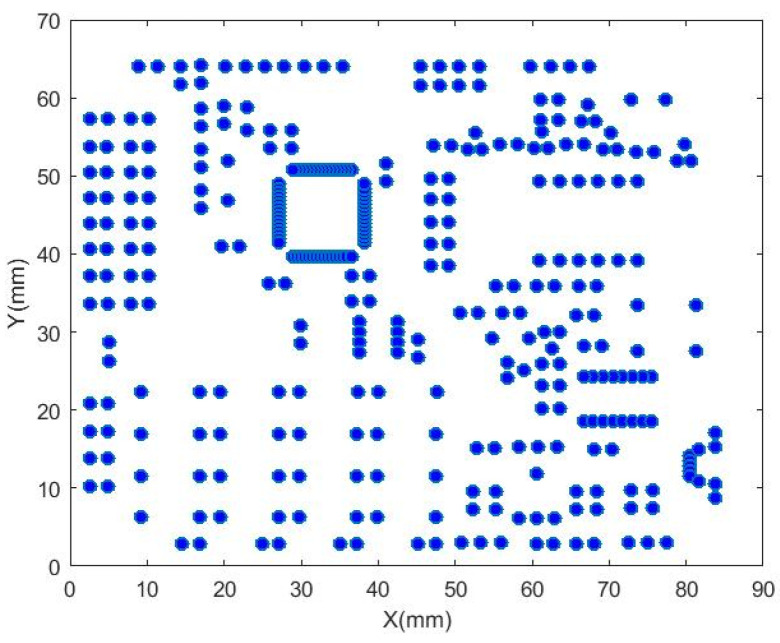
Solder joint position data display.

Altium Designer software is used to export the solder joint position information file of the circuit board, and the obtained solder joint position information is shown in Table 4. After exporting the solder joint position information according to the circuit board source file, the data needs to be processed accordingly. The imported solder joint position data is displayed as shown in Figure 19.

**Figure 20:**
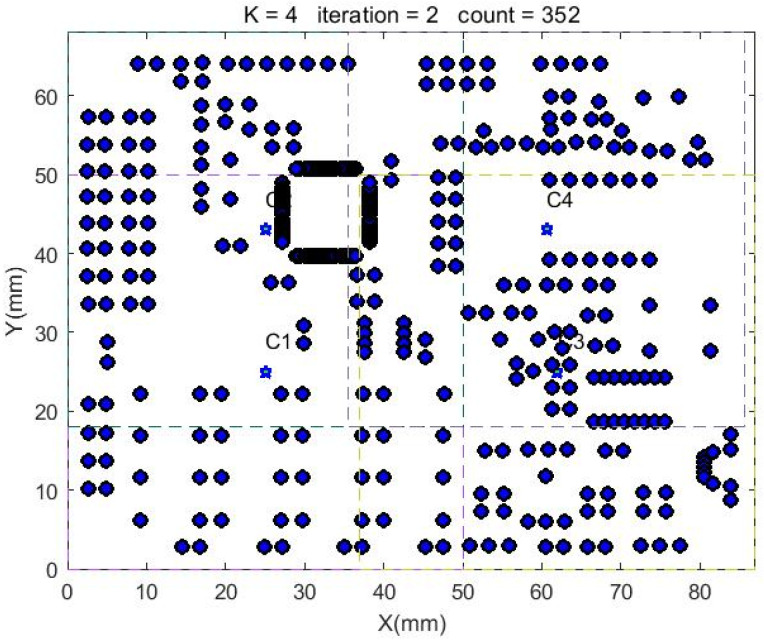
Circuit board processing frame.

In the improved clustering algorithm, the grid is divided into 4 × 3 grids to set the initial cluster center, and then the algorithm flow is started. When there are no merged processing frames, the algorithm stops and outputs the corresponding results. The number of processing frames generated by the improved clustering algorithm in the test circuit board is four, and the corresponding processing frames are generated as shown in Figure 20. The machining frame generated after testing can cover all solder joints, and the corresponding center points of each machining frame are shown in Table 5.

**Figure 21:**
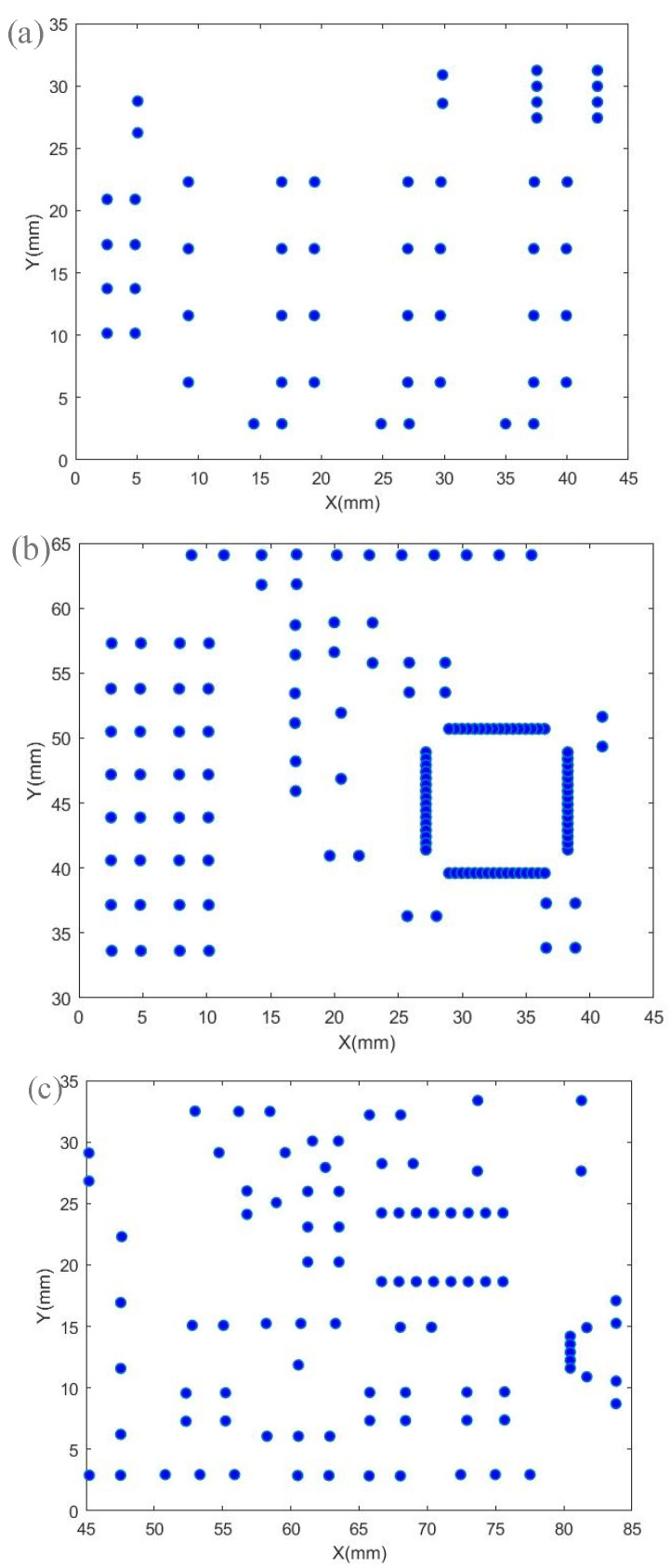

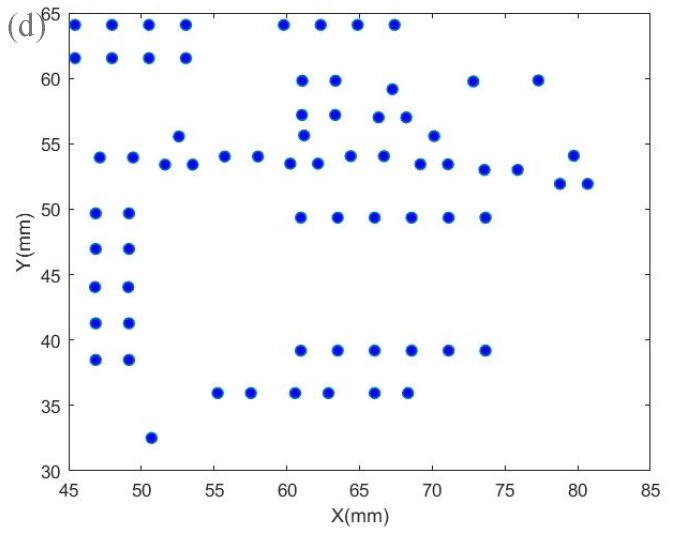
Distribution of solder joints belonging to each processing frame. (**a**) Processing frame 1, (**b**) Processing frame 2, (**c**) Processing frame 3, (**d**) Processing frame 4.

The solder joints in the processing frame are extracted to facilitate the next path optimization. The distribution of solder joints corresponding to each processing frame is shown in Figure 21. It has been verified that the solder joints in each processing frame do not exceed the processing range, each solder joint has a corresponding processing frame, and the generated processing frame can meet the requirements of system processing.

### 4.2. Analysis of Machining Path Optimization Algorithm

**Figure 22:**
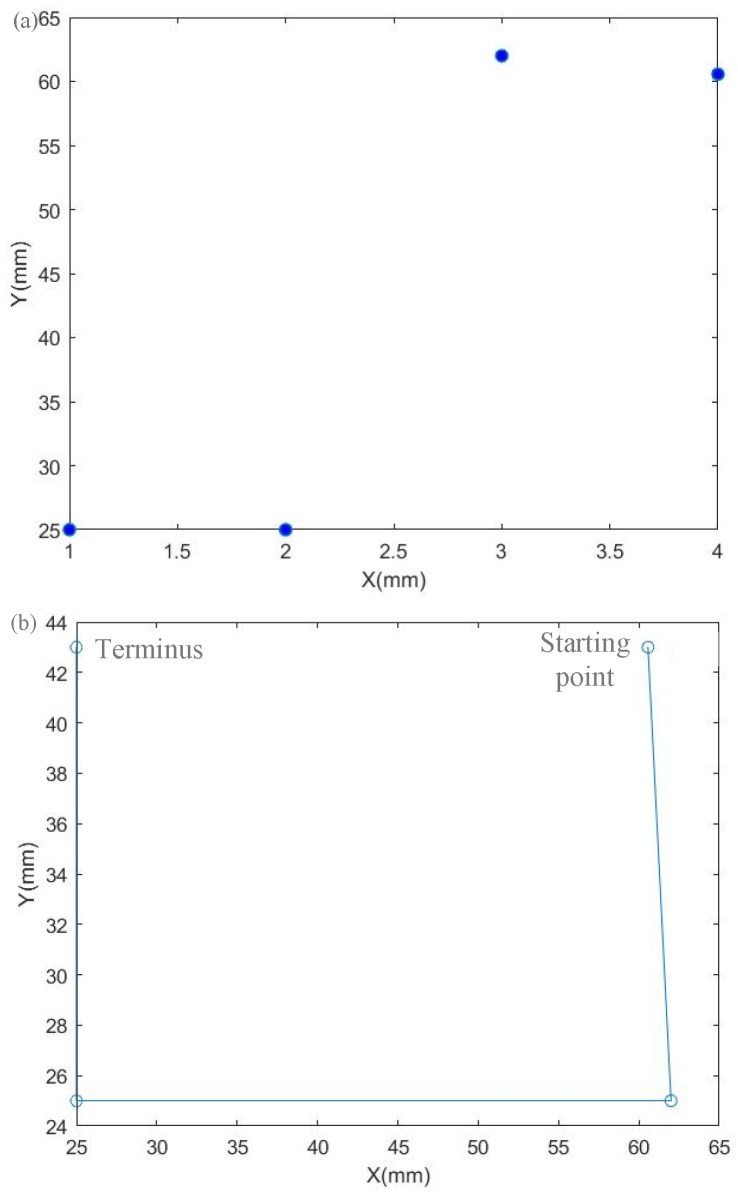
Optimization of the movement path between processing frames. (**a**) Processing frame center point distribution. (**b**) Processing frame center point optimization path.

Moving points between processing frames for path optimization can effectively reduce the movement path of the motion axis. Let the overall maximum number of iterations of the hybrid algorithm be the same as the maximum number of iterations of the comparative ant colony algorithm, and the maximum number of iterations is 150. The basic parameter settings of the ant colony algorithm in the hybrid algorithm are the same as those of the comparative ant colony algorithm, α=1, β=5, ρ=0.1, and the number of ants is 50. The basic parameters of the particle swarm algorithm in the hybrid algorithm are set as $c_{1}=0.5,\;c_{2}=0.5$, and the number of particle swarms is 50. Due to the small number of moving points between the processing frames, the advantages of each algorithm cannot be well reflected. As shown in Table 6, it can be concluded that the optimal paths of the two algorithms are the same, and the running time of the hybrid algorithm is shorter. Figure 22 shows the effect of optimizing the movement path of the machining frame using the hybrid algorithm.

The laser needs to process the solder joints along the corresponding path in each processing frame; after the processing frame path planning is completed, the laser beam movement path in each processing frame area needs to be planned. In the test, the ant colony algorithm and the hybrid algorithm are used for path planning, and the algorithm parameter settings are the same as the above. The comparison results are shown in Table 7.

**Figure 23:**
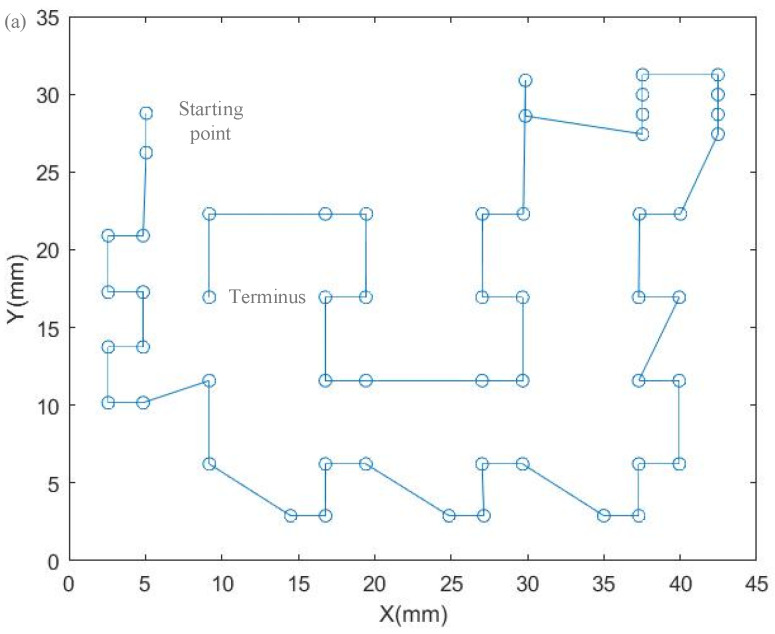

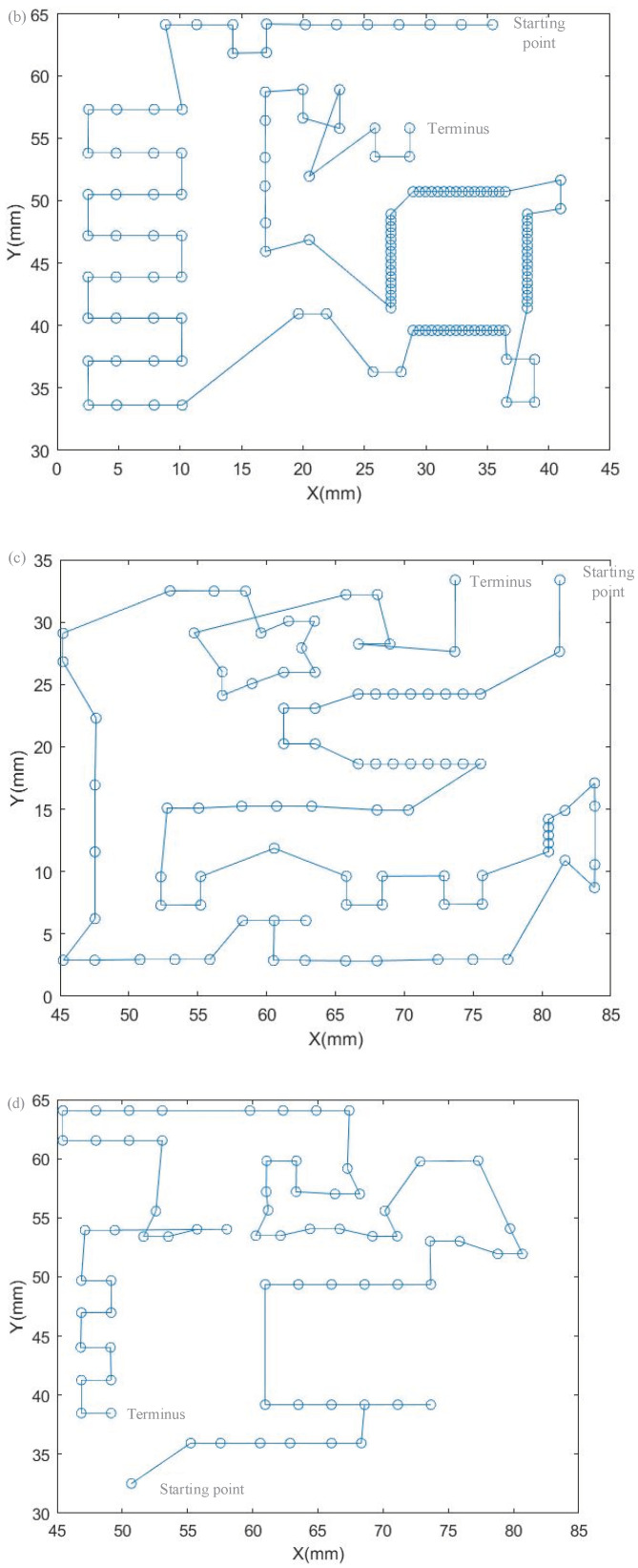
Optimization of the solder joint path in the processing frame. (**a**) Processing frame 1, (**b**) Processing frame 2, (**c**) Processing frame 3, (**d**) Processing frame 4.

Compared with the ant colony algorithm, the hybrid algorithm has better solution accuracy and shorter running time during the solution process. The hybrid algorithm is used in the system to plan the solder joints in each processing frame area, as shown in Figure 23.

## 5. Conclusions

In this paper, a galvanometer scanning laser soldering system is established, and the camshaft and workpiece are calibrated according to the characteristics of the system. At the same time, the problem of path planning in the soldering process of the system is analyzed. Aiming at the problem of low efficiency of ant colony algorithm in path planning, a path optimization algorithm with full coverage time of solder joints was proposed. The algorithm used the particle swarm optimization algorithm to optimize the initial pheromone of the ant colony, combined with the system characteristics and K-means clustering algorithm to generate a processing framework covering all solder joints. At the same time, the hybrid algorithm is used to optimize the moving path of the motion axis between the processing frames and the scanning moving path between the galvanometer and the soldering point in the processing frame area. The simulation and experimental results show that the algorithm can meet the needs of soldering path planning well. In the future, the fusion method and fusion time of the algorithm will be further studied.

## Figures and Tables

**Table 1:** Parameters of the vibrating lens.

Category	Parameter	Category	Parameter
Maximum scan angle	±20°	Working temperature	25 ± 10 °C
Effective scanning angle	±12.5°	peak current (A)	20
Repeatability (uRad)	<8		

**Table 2:** Parameters of semiconductor laser.

Category	Parameter	Category	Parameter
Center wavelength	915 nm	Fiber core diameter	400 μm
Output power	0~100 W	Cooling method	TEC cooling/water cooling
Package form	Fiber coupling		

**Table 3:** Thermometer technical parameters.

Category	Parameter	Category	Parameter
Temperature range	100–600 °C	Spectral range	2.3 μm
Temperature resolution	0.1 °C	Response time	1 ms

**Table 4:** Solder joint location.

Serial Number	X	Y
1	58.2536	6.0662
2	60.5536	6.0662
3	62.8536	6.0662
⋮	⋮	⋮
350	77.5208	2.9464
351	74.9808	2.9464
352	72.4408	2.9464

**Table 5:** The position of the center point corresponding to the processing frame.

Processing Box Number	Source Center
1	(25,25)
2	(25,43)
3	(62,25)
4	(60.5678,43)

**Table 6:** Comparison of moving path optimization algorithms between processing frames.

	Ant Colony Algorithm	Mixed Algorithm
Optimization path (mm)	108.6246	108.6246
Running time (s)	0.46002	0.42249

**Table 7:** Algorithm comparison.

Numbering	Ant Colony Algorithm		Mixed Algorithm	
	Optimization Path (mm)	Running Time (s)	Optimization Path (mm)	Running Time (s)
1	208.5278	3.7432	213.4559	2.7804
2	283.593	17.9577	275.7613	15.7682
3	291.971	13.4041	293.108	9.8753
4	216.7478	8.4758	219.5227	4.1466

## Data Availability

Data sharing not applicable.
